# Bone Mineral Density and First Line Imaging with [^18^F]fluorocholine PET/CT in Normocalcemic and Hypercalcemic Primary Hyperparathyroidism: Results from a Single Center

**DOI:** 10.3390/diagnostics14222466

**Published:** 2024-11-05

**Authors:** Dagmar Schaffler-Schaden, Gregor Schweighofer-Zwink, Lukas Hehenwarter, Antje van der Zee-Neuen, Maria Flamm, Mohsen Beheshti, Christian Pirich

**Affiliations:** 1 Institute of General Practice, Family Medicine and Preventive Medicine, Center for Public Health and Healthcare Research, Paracelsus Medical University, Strubergasse 21, 5020 Salzburg, Austria; 2 Department of Nuclear Medicine and Endocrinology, University Hospital Salzburg, Muellner Hauptstrasse 48, 5020 Salzburg, Austria; 3 Center for Physiology, Pathophysiology and Biophysics, Institute for Physiology and Pathophysiology, Paracelsus Medical University, Strubergasse 21, 5020 Salzburg, Austria; 4 Gastein Research Institute, Paracelsus Medical University, 5020 Salzburg, Austria

**Keywords:** primary hyperparathyroidism, [^18^F]fluorocholine PET/CT, bone mineral density, parathyroid adenoma, osteoporosis

## Abstract

**Objectives**: Primary hyperparathyroidism (PHPT) is associated with normal or elevated calcium levels and affects bone mineral density. The proportion of cases predisposed to metabolic bone disease is unknown in patients with PHPT. The aim of this study was to assess bone mineral density and bone quality in patients with normo- or hypercalcemic primary hyperparathyroidism undergoing baseline parathyroid gland assessment with [^18^F]fluorocholine PET/CT imaging. **Methods:** A total of 140 consecutive patients were enrolled in this observational study. All patients with normo- or hypercalcemic primary hyperparathyroidism underwent dual-energy X-ray absorptiometry (DXA) for assessment of bone mineral density (BMD) and trabecular bone score (TBS). [^18^F]fluorocholine PET/CT was performed in all patients for the detection and localization of parathyroid adenoma. Hyper- and normocalcemic patients were compared with regard to the proportion of osteoporosis and osteopenia, T-Score, TBS, serum calcium, phosphorus and parathyroid hormone levels, the maximum standardized uptake value (SUVmax) in PET/CT imaging, and laboratory results. **Results:** The majority of patients was female (88.57%) and had a pathologic bone mineral density (52.86%). Overall, 33 patients had osteoporosis and 41 osteopenia. The mean lumbar T-Score was −1.48 (SD 1.37) and the T-Score of the femoral neck was −1.21 (SD 0.92). Mean TBS was also decreased (−2.13). No difference was found between normo- or hypercalcemic patients regarding bone metabolism and imaging parameters. **Conclusions:** More than half of patients with normo- or hypercalcemic PHPT showed abnormal BMD. First-line [^18^F]fluorocholine PET/CT identified parathyroid adenoma in a high proportion of patients, even in patients with normocalcemic PHPT. The early evaluation of metabolic bone disease seems desirable in clinical management of females with PHPT.

## 1. Introduction

Primary hyperparathyroidism (PHPT) is characterized by inappropriately high levels of parathyroid hormone (PTH). The most common cause of PHPT is solitary adenoma of the parathyroid gland (80%), which occurs approximately four times more often in women than in men [1]. Symptomatic PHPT with nephrolithiasis and fractures is rare today due to routine osteoporosis and laboratory screening. Nowadays, most patients are oligosymptomatic or do not have any symptoms at all [2]. Osteoporosis is highly prevalent in patients with PHPT, and loss of bone occurs predominantly at cortical sites [3,4]. Many individuals suffer fragility fractures with a bone mineral density (BMD) in the osteopenic or even normal range, revealing substantial insufficiency of BMD measurement alone. Recently, the use of the trabecular bone score (TBS) as a predictor of fracture risk was suggested in patients with PHPT [5]. The trabecular bone score (TBS) reflects bone microarchitecture and is a parameter for bone quality. Thus, a low TBS can predict increased fracture risk independently of BMD [6]. Most patients with PHPT have elevated calcium levels, and normocalcemic PHPT (nPHPT) is less commonly diagnosed. NPHPT is characterized by a normal serum calcium concentration and elevated PTH levels after the exclusion of secondary causes for PHPT [7]. The prevalence of nPHPT depends on the population observed and is a subject of ongoing debate. The epidemiology and natural history of nPHPT are still conflicting, and the numbers reported vary substantially between 0.4 and 8.9% [8]. According to the Fourth International Workshop for the Management of Asymptomatic PHPT, nPHPT is recognized as a phenotype of PHPT, with some patients developing hypercalcemic PHPT (hPHPT) [9,10].

The current treatment of choice for all patients with PHPT is minimally invasive parathyroidectomy (PTX) with intraoperative parathyroid hormone monitoring [11]. Following the American Association of Endocrine Surgeons Guidelines, indications for PTX are previous fragility fractures or osteoporosis, but even asymptomatic patients reported better quality of life after resection [12]. Patients who undergo surgery have a better prognosis than those treated conservatively [13]; however, higher age and comorbidities may be reasons for non-surgical treatment [14]. After PTX, BMD also seems to increase in patients with nPHPT [15]. It is therefore assumed that fracture risk decreases after PTX, but larger randomized controlled clinical trials (RCTs) are currently lacking and study results are controversial [16]. For patients with PHPT who do not undergo surgery, the regular monitoring of BMD and blood counts is recommended [17]. To avoid unnecessary and unsuccessful surgery, the precise preoperative localization of parathyroid glands is important. Cervical ultrasound combined with [^99m^Tc]Tc-MIBI scintigraphy is most often used to assess parathyroid disease with a sensitivity of up to 95% [18,19]. Accurate preoperative localization of glands in patients with PHPT will become more difficult in the future due to the increasing prevalence of asymptomatic cases with small adenomas or only mild hyperfunction. According to the EANM practice guidelines for parathyroid imaging, [^18^F]fluorocholine PET/CT should be considered even for patients with negative standard imaging [20]. Patients with nPHPT often have smaller adenomas, which probably contributes to the lower sensitivity of preoperative imaging in these patients [21]. Recent study results revealed that [^18^F]fluorocholine PET/CT is superior in identifying parathyroid adenomas compared to [^99m^Tc]Tc-MIBI scintigraphy, and is thus suggested as the preferred imaging modality for the preoperative localization of parathyroid glands [22,23]. [^18^F]fluorocholine PET/CT is also advantageous in identifying concomitant nodular goiters and in detecting atypically located adenomas [24]. The use of PET/CT in nPHPT has rarely been described previously and the study cohorts in these studies were small [25]. However, the proportion of patients with lowered bone mineral density is unknown in patients with primary hyperparathyroidism undergoing a workup with more sensitive molecular imaging techniques. This study explores several aspects of primary hyperparathyroidism: initially, this study examines disparities in bone density between nPHPT and hPHPT, followed by an analysis of the PET/CT detection rates in both groups by correlating the imaging findings with the surgical outcomes.

## 2. Methods

**Figure 1:**
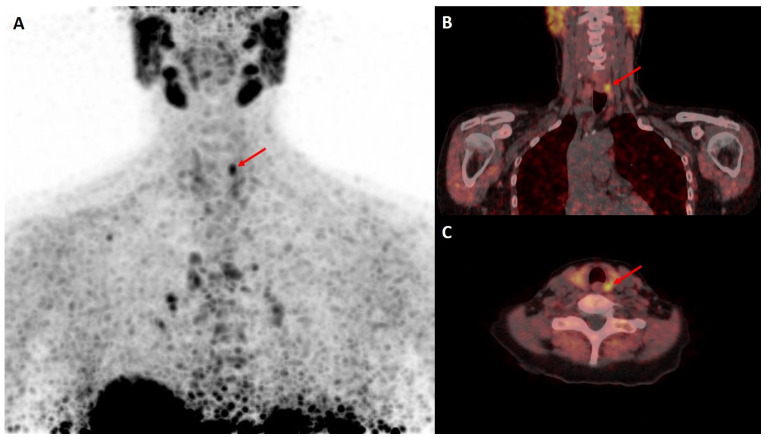
Imaging of a parathyroid adenoma, indicated by a red arrow, in a 74 year old female patient with elevated calcium levels using [^18^F]fluorocholine PET/CT, showing (**A**): maximum-intensity-projection PET, (**B**): coronal PET-CT and (**C**): transaxial PET-CT slices.

**Figure 2:**
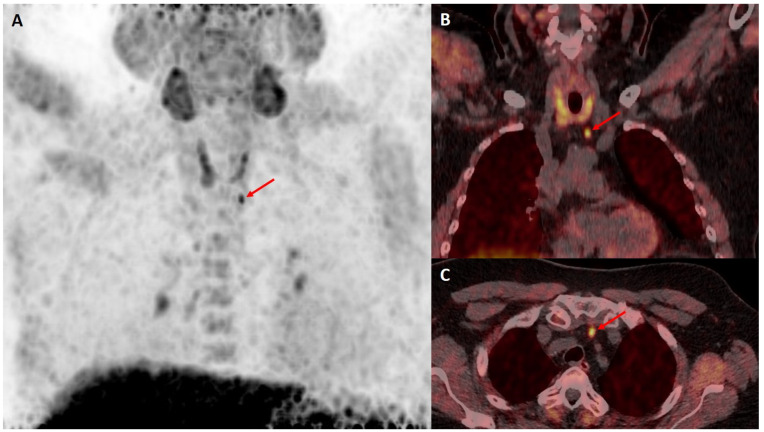
Imaging of a parathyroid adenoma, indicated by a red arrow, in a 65 year old female patient with normal calcium levels using [^18^F]fluorocholine PET/CT, showing (**A**): maximum-intensity-projection PET, (**B**): coronal PET-CT and (**C**): transaxial PET-CT slices.

In this prospective cohort study, we included patients with primary hyperparathyroidism and normal (<2.63 mmol/L, corrected for albumin) or elevated calcium levels (≥2.63 mmol/L, Table 1) and elevated levels of PTH (>65 pg/mL) attending the University Clinic of Nuclear Medicine and Endocrinology in Salzburg for screening or follow-up of osteoporosis by dual-energy X-ray absorptiometry (DXA) between 2015 and 2019. During that period, individuals underwent an imaging workup consisting of cervical ultrasound and a consecutive [^18^F]fluorocholine PET/CT (Figure 1 and Figure 2). There was no conventional [^99m^Tc]Tc-MIBI scintigraphy or [^99m^Tc]Tc-tetrofosmin parathyroid scintigraphy included in the workup of our patients. All patients underwent neck sonography using a standardized approach as recommended by the Austrian Thyroid Association using Siemens Acuson NX3 equipped with linear VF 12-4 and 16-5 transducers. Doppler ultrasound was applied, showing characteristic peripheral extrathyroidal feeding arteries and internal vascularity. Patient workup followed DVO guidelines, and all individuals eligible for surgery underwent assessment by an expert panel including specialists in internal medicine, nuclear medicine, and endocrine surgery. The 25 OH-vitamin D3 level was assessed in all individuals of the study population. All individuals with a detectable cause of secondary hyperparathyroidism (e.g., vitamin D deficiency, malabsorption, kidney disease or Familial Hypocalciuric Hypercalcemia) or on medication with lithium or thiazides were excluded.

### 2.1. Assessment of Bone Mineral Density

All patients received a detailed questionnaire to assess the risk of osteoporosis, which included items about family history, medication usage, comorbidities, previous fractures, alcohol and tobacco intake and, for female participants, their gynecological history. Hologic Discovery QDR 4500, Bedford, MA, USA was used for all DXA scans of the lumbar spine, femoral neck, and total hip. The scan measurements and analyses were conducted following the standard procedures: TBS was extracted from lumbar spine DXA and evaluated using TBSiNsight© software, version 3.0.2.0., Med-Imaps, Bordeaux, France. Measurements of BMD were performed at three sites in the same session and with the same DXA device: lumbar spine (L1–L4), total hip, and femoral neck. A T-score of ≤−2.5 standard deviation (SD) at any location was considered diagnostic of osteoporosis. TBS thresholds were defined as the standard deviation related to the respective T-Score values; TBS ≥ −1 was considered as normal.

### 2.2. [^18^F]fluorocholine PET/CT Imaging Protocol

A Philips Ingenuity TF (Philips Healthcare, Veenpluis 6, 5684 PC Best, The Netherlands) was used in 140 patients. Imaging was performed 60 min after intravenous injection of 200 MBq [^18^F]fluorocholine. PET/CT acquisitions were obtained from the base of the skull to the diaphragm to include possible ectopic adenomas. Images were reconstructed using a three-dimensional ordered-subsets iterative time-of-flight (BLOB-OS-TF) algorithm after correction for scatter and attenuation. For attenuation and anatomical correlation, we used a low-dose CT scan (50 mA, 120 kV, collimation 64 × 0.625 m^2^, slice thickness 3 mm, and reconstruction increment 1.5 mm). Images were read separately by an experienced nuclear medicine physician and a radiologist using advanced PET/CT review software (Philips IntelliSpace, version 8.0, for the Philips Medical Systems scanner), which allowed simultaneous scrolling through the corresponding PET, CT, and fusion images in the transverse, coronal, and sagittal planes. False-positive reactive lymph nodes were excluded on the basis of CT anatomy and location. Lesions were localized anatomically to six regions: right upper, right lower, left upper and left lower thyroid, intrathyroidal, and ectopic (e.g., mediastinal). The thyroid gland showed mild to moderate physiological tracer uptake on [^18^F]fluorocholine PET/CT, but this did not affect the interpretation of abnormal parathyroid lesions, especially when assessing transaxial images. For semiquantitative analysis, the maximum standardized uptake value (SUVmax) was calculated by using an automated 3D-VOI tool with a 50% cut-off of maximum SUV. The maximum length of the metabolic diameter of the pathological parathyroid lesions was measured manually within the provided review software. Neck ultrasonography was performed in all patients for morphological correlation and the improvement of image interpretation.

### 2.3. Statistical Analysis to Address the Study Aim

Demographic and clinical patient characteristics were described according to their metric properties for the total study population (*n* = 140). Pairwise correlations between T-scores and PTH levels were calculated. Subsequently, normo- and hypercalcemic patients were compared with regard to T-Score FN, T-Score LS, TBS, SUV max, osteoporosis, osteopenia, PTH pg/mL, Ca mmol/L, and P mmol/L using chi-square tests for categorical variables and independent sample *t*-tests for continuous variables.

To explore whether histologically confirmed adenoma was associated with increased odds of osteoporosis or osteopenia, a variable was first computed distinguishing between patients without or with pathology (i.e., osteoporosis or osteopenia). Next, multivariable logistic regression was computed exploring the association of histologically confirmed adenoma with the odds of pathology while adjusting for age, sex and calcium levels. All analyses were conducted using STATA 12.0 (SE). Statistical significance was assumed as *p* ≤ 0.05.

## 3. Results

The study population encompassed 140 individuals. The majority of patients were female (88.6%). Forty-three patients were normocalcemic, but most patients had elevated calcium levels (67.7%). Overall, 87 patients underwent surgery, but for 2, no detailed surgical data were available. Seventy-seven had histologically confirmed adenoma or hyperplasia (n = 4). Fifty-three patients had no surgery due to different reasons (e.g., refusal or comorbidities). All patients underwent screening for osteoporosis, 21 patients suffered prior fracture (n = 120; 17.5%), and 9 patients received antiresorptive treatment (n = 120; 7.5%).

The majority of the study population had a pathologic BMD (52.86%). Overall, 23.57% had osteoporosis and 29.29% had osteopenia (Table 1).

Correlation of T-scores with PTH levels revealed no significance (LWS *p* = 0.88, SH *p* = 0.51, respectively). Patients with nPHPT and hPHPT had a decreased mean TBS. (Table 2).

Multiglandular disease was apparent in one patient with nPHPT and six patients with hPHPT. Normocalcemic patients underwent surgery more often (62.79% and 58.89%, respectively). Overall, 94.3% of patients who underwent surgery had a correct match in the PET/CT imaging. Four patients had positive imaging without corresponding findings in surgery (4.6%), three of whom were hypercalcemic, and one had missing calcium values. However, two of these patients had normal Ca and PTH values at the follow-up 4 months after surgery. A total of 27 patients with nPHPT had successful surgical resection after imaging with [^18^F]fluorocholine PET/CT (100% match in nPHPT).

No significant difference in BMD was found regarding surgery (Table 3). Patients with confirmed adenoma had (non-significantly) higher odds of osteoporosis or osteopenia compared to those without it (OR 1.82 [95% CI 0.69; 4.79]), independent of age, calcium levels, and sex. There was no significant difference in SUVmax in relation to calcium levels or osteoporosis and osteopenia (7.77 (±2.66) vs. 9.00 (±3.76); *p* = 0.122).

## 4. Discussion

To the best of our knowledge, this is the first study to investigate the proportion of patients with either osteopenic or osteoporotic bone mineral density comparing normocalcemic with hypercalcemic PHPT-patients. Interestingly, the proportion of osteoporosis as assessed by standardized DXA measurement was independent from whether patients had normo- or hypercalcemia. These results might influence the clinical strategy and support performing highly sensitive PET/CT-imaging early in the workup of hyperparathyroidism. In our cohort, [^18^F]fluorocholine PET/CT showed high overall accuracy (94.3%) in localizing the parathyroid glands.

Notably, most patients in our cohort had pathologic BMD. Individuals with surgically confirmed adenoma or hyperplasia had higher odds of osteoporosis or osteopenia. Regarding BMD, no significant difference was found between normocalcemic and hypercalcemic patients. As Yan et al. have already reported, levels of calcium do not correlate with the rate of osteoporosis [26]. The estimated prevalence of osteoporosis in PHPT varies substantially between 39 and 62.9% [3], while another study including patients with nPHPT reported a prevalence of 53.3% [27]. The prevalence of osteopenia and osteoporosis in our study sample was 29.29% and 23.57%, respectively. Previous studies including subjects with comparable mean age have shown higher prevalence in the general population [28]. The role of TBS in PHPT is not clear yet. As in patients without hyperparathyroidism, a low TBS reflects a change in bone microarchitecture and was reported to be useful in predicting fracture risk in patients with PHPT [29]. On the contrary, in a cohort of Romanian patients with PHPT, a low TBS was not associated with vertebral fractures [30]. Studies on TBS in patients with nPHPT are scarce. There was no difference in mean TBS between our groups; this is consistent with one other study, although the reported sample size is small (only six patients with nPHPT [31]).

Nowadays, PHPT often presents asymptomatically. As we were able to show in our cohort, the prevalence of osteoporosis is also high in patients with nPHPT. Therefore, it seems reasonable to examine asymptomatic persons to prevent future fragility fractures. However, the reported high rates of osteoporosis and/or osteopenia with the resulting risk of fracture require exact diagnostic management in patients with primary hyperparathyroidism. Although ultrasound (US) and [^99m^Tc]Tc-MIBI scintigraphy are the most widely used diagnostic methods, [^18^F]fluorocholine PET/CT shows higher accuracy, as a recent systematic review including 1112 patients reported [32]. [^18^F]fluorocholine PET/CT is particularly superior in detecting small and ectopic glands, which often occur in PHPT [22,33]. The superiority of [^18^F]fluorocholine PET/CT in the localization of parathyroid glands in nPHPT was reported earlier, but it is well known that the diagnostic localization of parathyroid glands in patients with nPHPT is particularly difficult. In our cohort, four patients who underwent surgery had an incorrect match (three of them had hPHPT, one value missing). All other patients with nPHPT had a correct match. Bossert et al. found that there was no significant difference in the detection rate of nPHPT and hPHPT patients, although the sample size was small (only seven patients in the nPHPT group [25]). Although functional imaging using PET/CT is a costly method, Quak et al. hypothesized in their work that higher costs of imaging might compensate for costs related to more extensive surgical exploration for adenoma localization when the standard imaging procedure is applied [34]. Cervical ultrasound is the most cost-effective imaging method in the diagnosis of hyperparathyroidism and avoids patient radiation exposure. However, the sonographic detection of parathyroid adenomas depends on the skills and experience of the operator and has an overall sensitivity between 55 and 87%, which is significantly low in nPHPT [35]. Moreover, the detection of glands can be challenging in the presence of goiters and large thyroid nodules or in ectopic glands [36]. In contrast to our previous findings and publications from other investigators [37,38], this study did not find a significant correlation between serum calcium and SUVmax. [^18^F]fluorocholine, which acts as the precursor to phosphatidylcholine, primarily reflects the biosynthesis of the cell membrane of the target organs. The intensity of [^18^F]fluorocholine, as measured by means of semi-quantitative PET analysis (e.g., SUVmax), is dependent on a number of factors, including the size, weight, and hormonal status (e.g., PTH in PHPT) of the target lesion, as well as its lipogenesis. This may also explain why an accurate SUV cut-off for differentiating between parathyroid adenoma and hyperplasia could not be established in previous studies. Furthermore, no significant difference was identified between SUVmax values in patients with osteoporosis and those with osteopenia. Hypercalcemia in PHPT is not solely a result of increased bone demineralization. Rather, it appears to be a multifactorial process.

Multiglandular disease was described more often in normocalcemic patients; in our cohort, we had only one patient with three adenomas in the nPHPT group (versus six patients in the other group) [39].

Reports about the benefit of PTX on fracture risk in PHPT are conflicting. Some authors suggest that successful PTX is associated with a significant gain in BMD in hypercalcemic and normocalcemic patients [40], but there are also contrary findings [41]. While some authors even recommend surgery for asymptomatic patients, a recent systematic review does not support the priority of PTX in patients with mild asymptomatic PHTP in terms of fracture risk [11,16]. Due to technical advances, it is now possible to perform PTX as a minimally invasive outpatient surgery. Compared to the bilateral open neck exploration, this procedure is less risky regarding postoperative hypocalcemia and laryngeal nerve injury [42]. Apparently, many patients do not undergo curative surgery despite their symptoms; this seems more likely for the group with normal calcium levels [26]. To date, it is unknown whether earlier intervention against PHPT will translate into improved long-term clinical outcomes with regard to bone mineral density. However, PTX is the only curative therapy and might be a recommendation for asymptomatic patients. Age should no longer be an argument to exclude patients with PHPT from minimally invasive surgery [11]. Since minimally invasive PTX is regarded as the standard of care, the exact preoperative localization of parathyroid glands is required to enable surgeons to safely locate the lesion within the smallest possible surgical field and avoid collateral damage through the expansion of the operation. In our cohort, we demonstrated that a substantial number of patients with normocalcemic primary hyperparathyroidism (nPHPT) underwent successful surgery following diagnostic imaging with [^18^F]fluorocholine PET/CT.

## 5. Conclusions

Osteoporosis is highly prevalent among patients with an initial diagnosis of PHPT. This also relates to patients with normocalcemic PHPT. [^18^F]fluorocholine PET/CT also has high detection rates in patients with nPHPT. Precise preoperative localization of the parathyroid glands enables minimally invasive surgical resection, even in patients with nPHPT. The benefit and cost-effectiveness of the standard use of advanced imaging techniques like [^18^F]fluorocholine PET/CT is dependent on the availability and access to these technologies and should be further studied.

## Figures and Tables

**Table 1:** Demographic characteristics of the study population (n = 140).

Age (mean, SD; range)	64.54 (11.96; 21 to 87)
Female, n (%)	124 (88.57)
hPHPT, n (%)	90 (67.7)
Osteoporosis; n (%)	33 (23.57)
Osteopenia; n (%)	41 (29.29)
TBS	−2.13 (1.42; −6.2 to 1.8)
T score LS	−1.48 (1.37; −4.70 to 2.90)
T score FN	−1.21 (0.92; −3.40 to 1.30)
PTH pg/mL	123.58 (77.74; 66 to 361)
Ca mmol/L	2.70 (0.18; 2.10 to 3.43)
P mmol/L	0.84 (0.18; 0.25 to 1.51)

n/hPHPT = normo-/hypercalcemic hyperparathyroidism; T-Score FN = femoral neck; T-Score LS = lumbar spine; TBS = trabecular bone score (without units); PTH = parathormone; Ca = calcium; P = phosphate.

**Table 2:** Comparisons of characteristics of normo- and hypercalcemic patients.

	Normocalcemic PHPT	Hypercalcemic PHPT	*p*
TSCORE FN; mean (SD)	−1.12 (0.96)	−1.24 (0.90)	0.51
TSCORE LS; mean (SD)	−1.46 (1.30)	−1.50 (1.42)	0.88
TBS; mean (SD)	−2.11 (1.59)	−2.11 (1.33)	0.98
SUV max; mean (SD)	8.31 (3.63)	8.42 (3.24)	0.86
Osteoporosis; (%)	25.58	24.44	0.89
Osteopenia; (%)	34.38	44.12	0.36
PTH pg/mL; mean (SD)	112.487 (SD 64.53)	128.13 (SD 83.21)	0.29
Ca mmol/L; mean (SD)	2.52 (SD 0.12)	2.78 (SD 0.14)	0.00
P mmol/L; mean (SD)	0.89 (SD 0.21)	1.11 (SD 2.79)	0.62

T-Score FN = femoral neck; T-Score LS = lumbar spine; TBS = trabecular bone score (without units); SUV max = standard uptake value maximum; PTH = parathormone; Ca = calcium; P = phosphate.

**Table 3:** Comparison of patients with regard to surgery.

	N = 140	No Surgery (n = 53)	Surgery (n = 87)	*p*
Age, mean (SD; range)	64.54 (11.97; 21 to 87)	68.02 (12.66; 21 to 84)	62.41 (11.07; 28 to 87)	0.01
Female, n (%)	124 (88.57)	47 (88.68)	77 (88.51)	0.91
hPHPT, n (%)	90 (64.29)	34 (64.15)	53 (60.92)	0.95
Osteoporosis, n (%)	33 (23.57)	11 (20.76)	22 (25.29)	0.54
Osteopenia, n (%)	41 (29.29)	12 (22.64)	29 (33.33)	0.10
TBS, mean (SD; range)	−2.13 (1.42; −6.2 to 1.8)	−2.07 (1.46; −6.20 to −0.10)	−2.17 (1.40; −5.70 to 1.80)	0.72
T score LS, mean (SD; range)	−1.48 (1.37; −4.70 to 2.90)	−1.11 (1.46; −4.40 to 2.90)	−1.72 (1.27; −4.70 to 1.50)	0.02
T score FN, mean (SD; range)	−1.21 (0.92; −3.40 to 1.30)	−1.26 (0.92; −3.40 to 1.30)	−1.17 (0.92; −2.80 to 0.80)	0.59
PTH pg/mL, mean (SD; range)	123.58 (77.74; 66 to 788)	115.10 (37.37; 66 to 224)	128.59 (93.65; 69 to 788)	0.34
Ca mmol/L, mean (SD; range)	2.70 (0.18; 2.10 to 3.43)	2.71 (0.16; 2.10 to 3.07)	2.70 (0.20; 2.19 to 3.07)	0.91
P mmol/L, mean (SD; range)	0.84 (0.18; 0.25 to 1.51)	0.83 (0.15; 0.47 to 1.16)	0.85 (0.19; 0.25 to 1.51)	0.57

T-Score FN = femoral neck; T-Score LS = lumbar spine, TBS = trabecular bone score (without units); SUV max = standard uptake value maximum; PTH = parathormone; Ca = calcium; P = phosphate.

## Data Availability

The data presented in this study are available upon request from the corresponding author.
